# Outcomes of pPCL Diagnosed Using the IMWG 2021 Consensus Definition: A Retrospective Multicenter Analysis

**DOI:** 10.3390/cancers18010177

**Published:** 2026-01-05

**Authors:** Priyanka Venkatesh, Razan Mansour, Yara Shatnawi, Akhil Jain, Christopher Strouse, Nausheen Ahmed, Muhammad Umair Mushtaq, Al-Ola Abdallah, Shebli Atrash, Barry Paul

**Affiliations:** 1 Division of Hematology and Oncology, The University of Alabama at Birmingham, Birmingham, AL 35294, USA; priyankavenkatesh@uabmc.edu; 2 Department of Medical Hematology and Oncology, Yale University, New Haven, CT 06510, USA; 3 Faculty of Medicine, Jordan University of Science and Technology, Irbid 22110, Jordan; 4 Division of Hematology, Oncology, and Blood & Marrow Transplantation, University of Iowa Hospitals and Clinics, Iowa City, IA 52242, USA; 5 Division of Hematologic Malignancies & Cellular Therapeutics, University of Kansas Medical Center, Westwood, KS 66205, USA; 6 Levine Cancer Institute, Atrium Health Wake Forest University School of Medicine, Charlotte, NC 28204, USA; shebli.atrash@advocatehealth.org

**Keywords:** plasma cell leukemia, stem cell transplant, plasma cell dyscrasias

## Abstract

Background: Primary plasma cell leukemia (pPCL) represents the most aggressive plasma cell dyscrasia with a poor prognosis and survival of <3 years. The International Myeloma Working Group (IMWG) adopted more inclusive diagnostic criteria for pPCL in 2021, including patients with 5% or more circulating plasma cells (down from 20%). Most published studies of pPCL do not include patients who meet the criteria for pPCL based on the newer diagnostic guidelines, and the data on the optimal treatment of pPCL is scarce. In our multi-center retrospective analysis, we report data on treatment regimens used in 67 pPCL patients to characterize outcomes in this population. Methods: We included patients with newly diagnosed pPCL between 2010 and 2023 based on the 2021 IMWG definition at one of three academic centers. Results: Our results suggest significant improvement in overall response rate (ORR) and progression-free survival (PFS) with the use of autologous stem cell transplant, but without additional benefit for a tandem transplant. The presence of high-risk cytogenetics was an independent risk factor for progression in the cohort. Conclusions: Our dataset represents one of the largest cohorts to date using the expanded definition of pPCL adopted by the IMWG in 2021 and stresses the importance of taking pPCL patients to transplant. Unfortunately, our study was not powered to determine the efficacy of individual induction and maintenance regimens, and many patients diagnosed with pPCL are ineligible for transplant based on end-organ damage at diagnosis or from disease that is refractory to induction therapy, underscoring the need for early diagnosis and treatment in hopes of preserving transplant eligibility.

## Simple Summary

Primary plasma cell leukemia (pPCL) is an aggressive plasma cell dyscrasia with an extremely poor prognosis. Recently, the definition of pPCL was broadened to include patients with fewer circulating plasma cells at diagnosis. We retrospectively examined outcomes in patients diagnosed with pPCL treated with novel therapies using the updated diagnostic criteria. Our data suggests that autologous stem cell transplant remains an effective treatment for these patients even after treatment with induction regimens containing novel therapies. However, our data also shows that a smaller proportion of pPCL patients undergo transplant compared to patients diagnosed with less aggressive plasma cell dyscrasias, most likely due to the aggressive nature of the disease. Our study underscores the need for rapid diagnosis and treatment in pPCL in hopes of preserving a patient’s transplant eligibility.

## 1. Introduction

Primary plasma cell leukemia (pPCL) is a rare and aggressive plasma cell dyscrasia that historically represents 2–4% of newly diagnosed multiple myeloma (MM) cases [1,2,3,4,5,6]. It is considered an ultra-high-risk disease that has a poor prognosis despite numerous advances in MM therapy over the last decade. Plasma cell leukemia has unique biological and clinical features that differentiate it from multiple myeloma [7]. Compared to MM, the karyotype of pPCL more frequently demonstrates hypodiploidy, chromosome 1 aberrations, and deletions of chromosome 17. These are all considered markers of adverse prognosis [8,9,10]. Furthermore, age ≥ 60 years, platelet count < 100, and circulating plasma cells > 20% have also previously been described as predictors of poorer survival [11]. The increased intrinsic genomic instability, presence of multiple adverse clinical and laboratory features, and high proliferative activity have all been cited as reasons for its poor prognosis [12].

Historically, the median overall survival in pPCL has been 4 to 11 months [3,4]. With the advent and widespread use of more novel therapies, including immunomodulatory agents and proteasome inhibitors, the OS improved to 1 year, and more patients were able to proceed to autologous stem cell transplant. Patients who received a stem cell transplant had a significant improvement in survival with a median PFS of 20 months and OS of 33 months [12,13,14,15,16].

In 2021, the International Myeloma Working Group (IMWG) adjusted the definition of plasma cell leukemia to include patients who have ≥5% circulating plasma cells, citing that these patients had a similar adverse prognostic impact as the previously defined PCL cutoff, which required patients to have >20% circulating plasma cells [17].

Prior studies in this population have largely described outcomes in patients meeting inclusion criteria according to the prior definition of pPCL. Given the lack of data to define appropriate treatment in pPCL based on the 2021 diagnostic guidelines, we conducted a multicenter retrospective analysis of patients with pPCL to determine optimum treatment for this condition using the more inclusive, newer diagnostic cutoff.

## 2. Methods

### 2.1. Patient Selection

We retrospectively and systematically reviewed 67 consecutive patients with a diagnosis of pPCL between 1 January 2010 and 31 December 2023 at Levine Cancer Institute, University of Iowa, or University of Kansas Medical Center who received at least 1 cycle of induction therapy.

### 2.2. Definitions

Primary plasma cell leukemia was defined using the 2021 IMWG guidelines as the presence of ≥5% circulating plasma cells in the peripheral blood. High-risk cytogenetics were defined as the presence of the following: del 17p, amplification (≥4 copies) of 1q, t (4;14), t (14;16), t (14;20), or complex cytogenetics. Response criteria were defined based on the 2012 IMWG consensus statement on plasma cell leukemia. PFS was defined as the time from diagnosis to relapse, progression, death, or last office visit. OS was defined as the time from diagnosis to death or the last office visit.

### 2.3. Statistical Analysis

Statistical analysis was performed using R Core Team (v2024) software. We reported continuous variables as mean (min, max) and median (IQR) and dichotomous factors as total numbers and frequencies. Fischer’s exact test was used to analyze contingency tables, and the Wilcoxon rank-sum test was used to compare independent samples. The International Myeloma Working Group (IMWG) criteria were used to determine responses to therapy. To estimate the relationship between variables and a time-to-event outcome, univariate/multivariate Cox proportional hazards analysis was used, which provides a hazard ratio and associated confidence interval (CI) for each variable with appropriate adjustments for censoring. Survival analysis to estimate PFS and OS was performed using Kaplan–Meier methods.

## 3. Results

### 3.1. Patient Demographics and Clinical Characteristics

We identified 67 patients consecutively diagnosed with pPCL. In our cohort, the median age was 64 years (range: 56–69 years). Fifty-one patients (76%) were Caucasian, and 15 patients (22.3%) were African American. A total of 43 patients (64%) were female, and 24 (36%) were male. About half the patients (35, 52%) had an ECOG performance status of 1 at diagnosis.

The median circulating plasma cells for the entire cohort was 33% (5, 82), of which 17 patients (25%) had 5–20% circulating plasma cells and thus would not have been defined as pPCL based on the prior definition of pPCL. 40 patients had high-risk cytogenetics, and extramedullary disease (EMD) was present in 16 (24%) patients. Most patients (N = 32; 48%) were categorized as R-ISS Stage III, 22 (33%) as Stage II, and 2 (3%) as Stage I, with 11 (16%) patients with missing data. The most commonly used induction regimen was cisplatin, etoposide, doxorubicin, and cyclophosphamide (PACE)-based chemotherapy (29 patients, 43%), followed by cyclophosphamide, bortezomib, and dexamethasone (CyBorD; 18 patients, 27%), and lenalidomide, bortezomib, and dexamethasone (VRd; 10 patients, 15%). Notably, daratumumab-containing quadruplet regimens were rarely used in our cohort (3 patients, 4%). Demographic data is summarized in Table 1 and was well balanced across all the characteristics studied. Demographic data comparing high-risk and standard-risk patients is shown in Supplementary Table S1.

### 3.2. Response to Treatment

Overall response rate (ORR) after induction therapy was 67% as shown in Table 2. Of the 29 patients who received PACE-based induction, 22 patients (76%) achieved ORR after induction. A total of 11 out of 18 patients (61%) received CyborD, 6 of 10 (60%) patients received VRd, 3 of 6 (50%) received triplet regimens, and 2 of 3 (67%) received quadruplet regimens achieved ORR. We are unable to comment on the comparative efficacy of induction regimens because of a lack of power.

Forty-six patients (69%) proceeded to stem cell transplant, with 17 patients undergoing a tandem transplant. In those patients who received a transplant, the ORR after induction therapy was comparable to the ORR after transplant (87% vs. 97%). Notably, the depth of response (defined as those patients who achieved at least a VGPR) significantly improved with transplant (78% vs. 54%). Thirty-four patients (51%) received maintenance chemotherapy post-transplant. Maintenance regimens used were very heterogenous, with 17 patients receiving triplet therapies, 9 patients receiving doublets, and 8 patients receiving single-agent maintenance.

### 3.3. Survival Statistics

**Figure 1:**
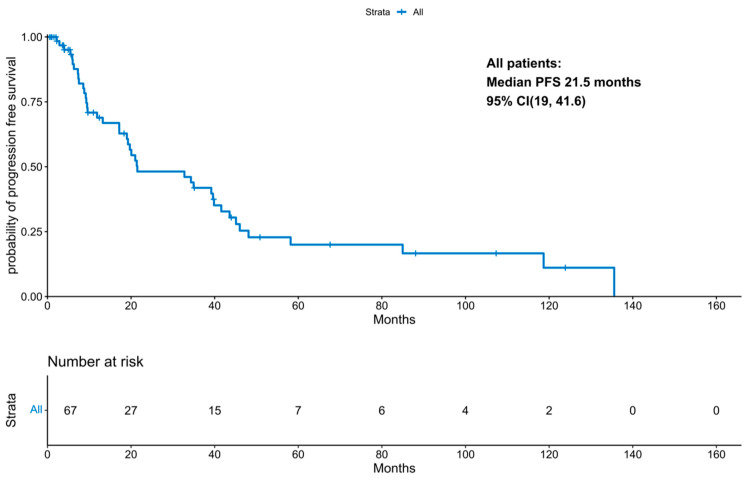
KM for progression-free survival for the entire cohort.

**Figure 2:**
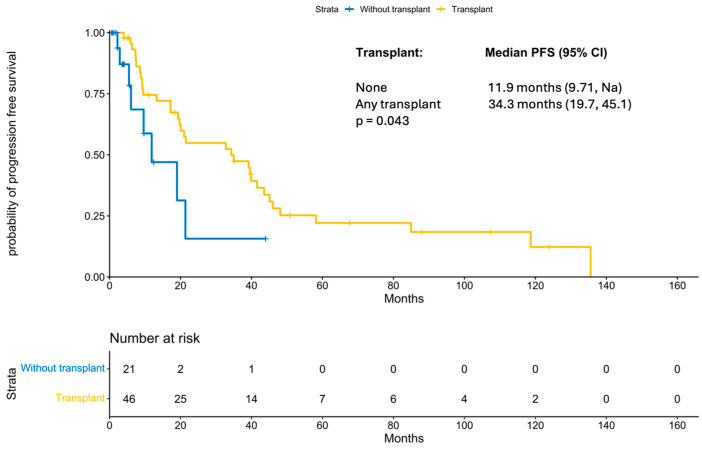
KM for progression-free survival by transplant status.

**Figure 3:**
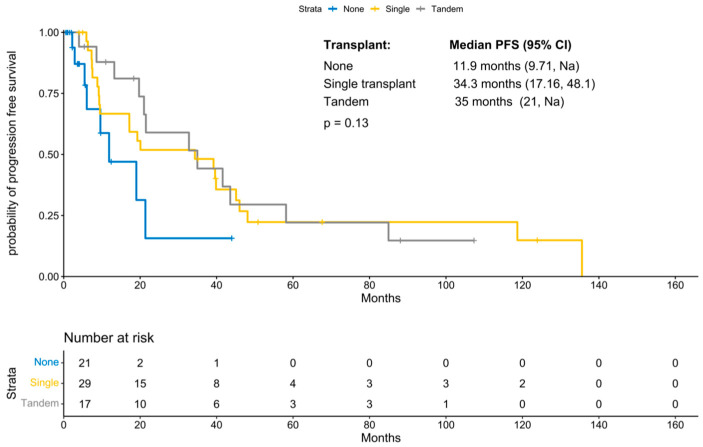
KM for progression-free survival by number of transplants.

With a median follow-up period of 131 months (IQR 21.47 months, NA), the median progression-free survival was 21.5 months (95% CI 19; 41.58 months; Figure 1). PFS improved significantly in those patients who underwent a transplant (11.87 months vs. 34.3 months, *p*-value: 0.04; Figure 2). Interestingly, patients who underwent a tandem transplant did not derive additional benefits compared to those who were transplanted once (PFS 34.97 vs. 34.32 months, *p*-value: 0.13; Figure 3).

Univariate/multivariate Cox proportional hazards analysis for the entire cohort showed that variables such as age, gender, performance status, R-ISS stage, and EMD status were not associated with PFS, as shown in Table 3. Undergoing any transplant improved PFS (HR 0.44; 95% CI 0.20, 0.99), as did achieving a response to transplant (HR 0.07; 95% CI 0.02, 0.27). High-risk cytogenetics was noted to be a risk factor for progression in univariate analysis of the entire population (HR 2.49; 95% CI 1.23, 5.02). Response to transplant (HR 0.07; 95% CI 0.02, 0.27) conferred a survival benefit in multivariate analysis.

In those patients who underwent transplant, high-risk cytogenetics was an independent risk factor for progression (HR 3.74; 95% CI 1.64, 8.55), as shown in Table 4. In a univariate analysis, response to transplant improved PFS (HR 0.05; 95% CI 0.01, 0.20), but the type of transplant, single versus tandem, did not influence PFS. Comparison of survival of the 17 patients meeting the new definition of pPCL with the cohort meeting the older definition showed no statistically significant difference in PFS (41.6 months vs. 20 months, HR 1.06, 95% CI 0.5–2.25, Supplementary Figure S1).

## 4. Discussion

Despite significant improvements in survival outcomes in MM owing to the widespread availability of novel agents and stem cell transplantation, pPCL has only seen a modest improvement in survival. A large retrospective study of 1357 patients by the Korean Myeloma Working Party, published after the IMWG adopted the newer diagnostic criteria in 2021, validated that, patients with 5–19% circulating plasma cells have a similar prognosis when compared to patients with >20% CPCs [18]. The European Myeloma Network expert panel consensus published in April 2025 recommended careful morphological evaluation and flow cytometry if needed to establish a diagnosis, especially in patients with around 5% CPCs. In our cohort, 25% of patients had CPCs between 5 and 20% and would have been excluded based on the prior definition [19]. Data guiding treatment decisions in pPCL are mostly based on retrospective studies that included patients defined using the older diagnostic criteria.

A Surveillance, Epidemiology, and End Results (SEER) database study of 445 patients with pPCL showed an improvement in median OS of patients diagnosed after 2006 from 5 months to 12 months [20]. The improvement in survival in these patients can likely be explained by the use of therapeutic agents such as lenalidomide, thalidomide, and bortezomib. There have been several retrospective analyses of patients treated with novel agents, which have shown improved response rates and survival when compared with conventional chemotherapy [11,15,21,22,23]. Furthermore, the use of transplant in combination with novel agents has been shown to provide additional survival benefit. The Greek Myeloma Study Group published real-world data in 2018 showing that patients treated with bortezomib had higher response rates than others, even in patients who were ineligible for transplant (ORR 70% vs. 58%) [24]. Another study by the same group in 2023 that included 110 patients with pPCL using the new definition showed significantly longer PFS in patients treated with VRd or daratumumab-based quadruplets [25]. In 2023, the phase II EMN12/HOVON-129 clinical trial prospectively studied the use of carfilzomib, lenalidomide, and dexamethasone (KRd) induction, +/− transplant, followed by KR maintenance in 61 pPCL patients [26]. Their results indicated that the use of a PI+IMiD (KRd) led to a large proportion of patients achieving VGPR or greater when compared to PI+chemotherapy. All patients in our cohort were treated with novel agent-based combinations that included a PI for induction. Although PACE-based induction regimens were most frequently used (43%), our study was not powered to determine the best induction regimen.

Regarding the use of transplantation in the treatment of pPCL, multiple prior studies have established that transplantation provides a survival benefit in pPCL. As discussed previously, Katodritou et al. reported a significantly improved PFS in patients treated with bortezomib+ASCT compared to others (18 months vs. 9 months, *p* = 0.004) [24]. Another recently published retrospective study by Singh et al., on 93 patients with pPCL, reported significant improvement in survival with transplant, irrespective of type [27]. The improved survival was also confirmed by the prospective EMN12/HOVON-129 clinical trial, which showed that the median PFS was 15.5 months in patients who underwent transplant and 13.8 months in those deemed ineligible for transplant [26]. Similarly, our study suggests a significant PFS benefit with transplant and shows that it is relevant even when using the expanded definition for pPCL.

Data from a European Society for Bone and Marrow Transplantation (EBMT) study that included 272 patients with pPCL who underwent autotransplants between 1980 and 2006 reported a median PFS of 14.3 months with improved PFS in those who achieved a CR after transplantation (HR, 0.64, CI, 0.39, 1.05) [28]. Univariate analysis of transplanted patients in our study similarly showed PFS benefit in patients who achieved response after transplant (HR 0.05, 95% CI 0.01–020, *p* < 0.001). The median PFS in our study was longer, likely owing to a lower proportion of patients with Stage III disease in our cohort (48% vs. 80%) and higher utilization of novel agents prior to transplant.

Lawless et al. undertook a retrospective analysis of 751 pPCL patients undergoing various consolidative transplant strategies in 2023 [29]. With a median follow-up period of 48.8 months, they reported a median OS of 33 months and a median PFS of 14 months, irrespective of transplant type. A total of 681 patients underwent an upfront autotransplant, and 70 patients received an upfront allotransplant, while 122 patients proceeded to a tandem transplant. When compared to single autotransplant, single allotransplant had a significantly higher risk for progression in the first 100 days due to NRM. However, tandem auto-allo had improved PFS after 100 days. The difference in PFS for tandem autotransplant versus single auto was not statistically significant. The OS at three years was not markedly different regardless of the strategy used. The Center for International Blood and Marrow Transplant Research (CIBMTR) published results from a retrospective study on 147 patients with pPCL receiving upfront transplants between 1995 and 2006, comparing auto- and allotransplants [30]. The OS at 3 years after autologous transplant was 64%, which demonstrated the safety and feasibility of consolidative transplant after initial induction therapy. They also reported a trend towards higher OS in patients who received tandem autotransplants. PFS at 3 years was comparable for the single vs. tandem transplant group (36% vs. 37%). As a follow-up to this study, CIBMTR reported a further analysis of 348 transplanted patients between 2008 and 2015 [16]. Despite the increase in transplant utilization, survival was reported to be inferior in this cohort overall, owing to the higher post-transplant relapse rates, which were thought to be due to more patients being eligible for transplant, lower use of maintenance therapy, and selection for more aggressive disease in this population. Prospective studies addressing transplant strategies in this cohort are limited. Results published by the Inter-group Francophone du Myelome (IFM) on 40 patients showed that tandem auto/auto plus maintenance therapy may lead to superior OS compared to auto/allo [31]. In our study, 17 patients (25%) underwent tandem transplant, and the median PFS was similar in those who underwent a single or tandem transplant (34.32 months versus 34.97 months). This data argues against the use of tandem transplants, given the lack of clear benefit and the additional toxicity associated with a second transplant.

Another consideration in treatment is the use of consolidation and maintenance therapy post-transplant. Gowda et al. published a retrospective study on 23 patients who underwent autotransplants for pPCL [14]. They reported that use of post-transplant maintenance was associated with longer PFS (16.9 vs. 3.9 months, *p* = 0.05). In our study, we show that PFS is improved in those patients who have a response to transplant and receive maintenance therapy, highlighting its prognostic significance. However, the type of maintenance therapies used in our cohort was very heterogeneous, limiting our ability to understand the benefit of specific maintenance regimens.

Various studies have examined the impact of cytogenetic abnormalities on outcomes in patients with pPCL [4,9,32]. Most recently, Diaz et al. reported the impact of high-risk cytogenetics on survival outcomes in 89 patients with more than 5% circulating plasma cells [33]. The OS of the entire population was 47 months (95% CI: 37, 100). When stratified based on high-risk cytogenetics, the median OS was 101 months (95% CI 57, NR) for those with no high-risk criteria versus 37 months (95% CI: 22, 63) for those with one or more high-risk cytogenetic abnormalities. In our study, we note that high-risk cytogenetics was an independent risk factor for progression in the transplant-only cohort (HR 3.74; 95% CI: 1.64, 8.55), indicating the need for universal testing for cytogenetic abnormalities for risk stratification and more aggressive upfront treatment in these patients.

Our study does have some important limitations. First, the sample size (67) and significant treatment heterogeneity did not provide adequate power to examine the efficacy of various induction and maintenance regimens. While our study found that PFS was longest in patients who underwent a transplant, this could be somewhat biased by the fact that fitter patients were preferentially transplanted, while frailer patients and those with significant comorbidities (some of which were likely a result of their pPCL) were unable to proceed to transplant. This may indicate that the patients who were able to undergo transplant in our cohort could have had clinically less aggressive disease. Furthermore, we were unable to report OS in our study due to significant censoring.

## 5. Conclusions

Our study shows that there is a trend towards improved progression-free survival in patients who undergo transplant in the context of the expanded definition for primary plasma cell leukemia. Patients who underwent any transplant had significantly improved PFS compared to those who did not undergo a transplant. When considering transplants in patients, high-risk cytogenetics was the only independent risk factor for progression. Response to transplant and maintenance therapy after transplant offered a significant PFS benefit in this population. As many patients diagnosed with pPCL are ineligible for transplant based on end-organ damage from uncontrolled disease or disease that is refractory to induction therapy, more efforts are needed to diagnose and treat pPCL to improve outcomes with induction therapy and to allow more patients to proceed to transplant.

## Figures and Tables

**Table 1:** Demographics summary.

Characteristic	N (%)
Age at Diagnosis (Median + IQR)	64 (56, 69)
Gender	
Male	43 (64%)
Female	24 (36%)
Race	
Caucasian	51 (76%)
African American	15 (22%)
Other	1 (2%)
PS	
0	11 (16%)
1	35 (52%)
2	14 (21%)
3	6 (9%)
Unknown	1 (2%)
Heavy Chain	
IgA	15 (22%)
IgG	30 (45%)
Non-secretory	3 (5%)
Light Chain only	19 (28%)
Light Chain	
Kappa	30 (45%)
Lambda	34 (51%)
Non-secretory	3 (5%)
Circulating Plasma Cells (%, Median + IQR)	33 (5, 82)
High Risk	
Yes	40 (60%)
No	26 (39%)
Unknown	1 (2%)
R-ISS Stage	
I	2 (3%)
II	22 (33%)
III	32.(48%)
Unknown	11 (16%)
EMD	
Yes	16 (24%)
No	51 (76%)

PS: Performance Status; ASCT: Autologous Stem Cell Transplant; R-ISS: Revised International Scoring System; EMD: Extra-Medullary Disease; IQR: Inter-Quartile Range; ORR: Overall Response Rate; NA: Not Available.

**Table 2:** Summary of response to therapy.

Characteristic	N (%)
ORR after Induction	
Yes	45 (67%)
No	9 (13%)
Unknown	13 (19%)
ORR after ASCT	
Yes	40 (60%)
No	4 (6%)
NA	23 (34%)
Type of Response after ASCT	
CR	26 (39%)
VGPR	10 (15%)
PR	4 (6%)
PD	4 (6%)
NA	23 (34%)
Maintenance post ASCT	
Yes	34 (51%)
No	9 (13%)
NA	24 (36%)

ASCT: Autologous Stem Cell Transplant; ORR: Overall Response Rate; NA: Not Available.

**Table 3:** Cox proportional hazards analysis for time to progression for the entire cohort.

Characteristic	Univariate Analysis			Multivariate Analysis		
	N	HR (95% CI)	*p*-Value	N	HR (95% CI)	*p*-Value
Age	67	0.99 (0.96 to 1.02)	0.67			
Gender	67		0.41			
Female		—				
Male		0.76 (0.38 to 1.49)				
PS	67		0.35	67		0.5
0		—			—	
1		0.70 (0.26 to 1.90)			0.69 (0.24 to 1.96)	
2		1.49 (0.51 to 4.31)			1.35 (0.42 to 4.30)	
3		1.37 (0.32 to 5.88)			0.54 (0.10 to 2.91)	
Unknown		0.67 (0.08 to 5.90)			0.75 (0.08 to 6.74)	
Circulating Plasma Cells (%)	65	1.00 (0.98 to 1.02)	0.68			
High-Risk Cytogenetics	67		0.03			
No						
Yes		2.49 (1.23 to 5.02)				
Unknown		0.00 (0.00 to Inf)				
Response to ASCT	67		<0.001	67		<0.001
No						
Yes		0.07 (0.02 to 0.27)			0.07 (0.02 to 0.26)	
NA		0.21 (0.06 to 0.78)			0.21 (0.04 to 0.98)	
R-ISS Stage	67		0.66			
1						
2		0.36 (0.05 to 2.93)				
3		0.53 (0.07 to 4.15)				
Unknown		0.44 (0.05 to 3.65)				
EMD	67		0.39	67		0.95
No						
Yes		1.36 (0.68 to 2.70)			1.03 (0.45 to 2.33)	
Type of Transplant	67		0.18			
None						
Single Transplant		0.46 (0.19 to 1.07)				
Tandem Transplant		0.42 (0.17 to 1.07)				
Transplant	67		0.04			
No						
Yes		0.44 (0.20 to 0.99)				
Maintenance Therapy	67		0.01			
No						
Yes		0.45 (0.16 to 1.25)				
NA		1.69 (0.58 to 4.89)				

HR: Hazard Ratio, CI: Confidence Interval, PS: Performance Status, ASCT: Autologous Stem Cell Transplant, R-ISS: Revised International Scoring System, EMD: Extra-Medullary Disease.

**Table 4:** Cox Proportional hazards analysis for time to progression for transplanted patients.

Characteristic	Univariate Analysis			Multivariate Analysis		
	N	HR (95% CI)	*p*-Value	N	HR (95% CI)	*p*-Value
Age	44	0.98 (0.95 to 1.01)	0.26			
Gender	44		0.61			
Female		—				
Male		0.82 (0.39 to 1.75)				
PS	44		0.33	44		0.5
0		—			—	
1		0.53 (0.19 to 1.46)			0.69 (0.24 to 1.96)	
2		1.07 (0.35 to 3.28)			1.35 (0.42 to 4.30)	
Unknown		0.54 (0.06 to 4.75)			0.54 (0.10 to 2.91)	
Circulating Plasma Cells (%)	42	0.99 (0.97 to 1.02)	0.5			
High-Risk Cytogenetics	44		0.003			
No						
Yes		3.21 (1.44 to 7.17)				
Response to ASCT	44		<0.001			
No						
Yes		0.05 (0.01 to 0.20)				
R-ISS Stage	44		0.32			
1						
2		0.24 (0.03 to 2.03)				
3		0.45 (0.06 to 3.57)				
Unknown		0.25 (0.03 to 2.31)				
EMD	44		0.44			
No						
Yes		1.36 (0.63 to 2.92)				
Type of Transplant	44		0.83			
Single Transplant						
Tandem Transplant		0.93 (0.45 to 1.90)				
Maintenance Therapy	44		0.003	44		<0.001
No						
Yes		0.41 (0.15 to 1.15)			13.2 (2.30 to 76.2)	
NA		7.99 (1.59 to 40.2)			0.39 (0.13 to 1.14)	

HR: Hazards Ratio, CI: Confidence Interval, PS: Performance Status, ASCT: Autologous Stem Cell Transplant, R-ISS: Revised International Scoring System, EMD: Extra-Medullary Disease.

## Data Availability

Data available on request from the authors/Supplementary Materials.

## Supplementary Materials

The following supporting information can be downloaded at: https://www.mdpi.com/article/10.3390/cancers18010177/s1, Table S1: Demographic table comparing high-risk and standard-risk patients; Figure S1: KM for progression free survival comparing old-pPCL and new-pPCL cohort.
