# Supplementary material for: Outcomes of pPCL Diagnosed Using the IMWG 2021 Consensus Definition: A Retrospective Multicenter Analysis
Source: Cancers (Basel). 2026 Jan 5;18(1):177. doi: 10.3390/cancers18010177 (PMC12785085; doi:10.3390/cancers18010177)
Supplement: Supplementary file 1 [file cancers-18-00177-s001.zip › cancers-3952269-supplementary.pdf]

Supplementary Table S1. Demographic table comparing high-risk and standard-risk patients.

**Characteristic**	**High-Risk**	**Standard Risk**	***p*-Value**	**q-Value**
**Age, Mean (SD)**	62 (10)	62 (10)	0.22	0.46
**Gender, n (%)**			0.34	0.47
Female	23 (58)	19 (73)		
Male	17 (43)	7 (27)		
**PS, n (%)**			0.25	0.46
0	3 (12)	8 (20)		
1	17 (65)	18 (45)		
2	4 (15)	10 (25)		
3	2 (7.7)	3 (7.5)		
Unknown	0 (0)	1 (2.5)		
**R-ISS, n (%)**			0.17	0.46
1	0 (0)	2 (5.0)		
2	13 (50)	9 (23)		
3	9 (35)	22 (55)		
NA	4 (15)	7 (18)		
**Circulating plasma cells, Median (IQR)**	30 (21 – 46)	30 (19 – 38)	0.29	0.47
**EMD, n (%)**	6 (23)	10 (25)	>0.99	>0.99
**Double refractory, n (%)**	16 (42)	6 (23)	0.21	0.46
**Triple refractory, n (%)**	12 (32)	1 (3.8)	0.012	0.26
**Penta refractory, n (%)**	5 (13)	0 (0)	0.15	0.46

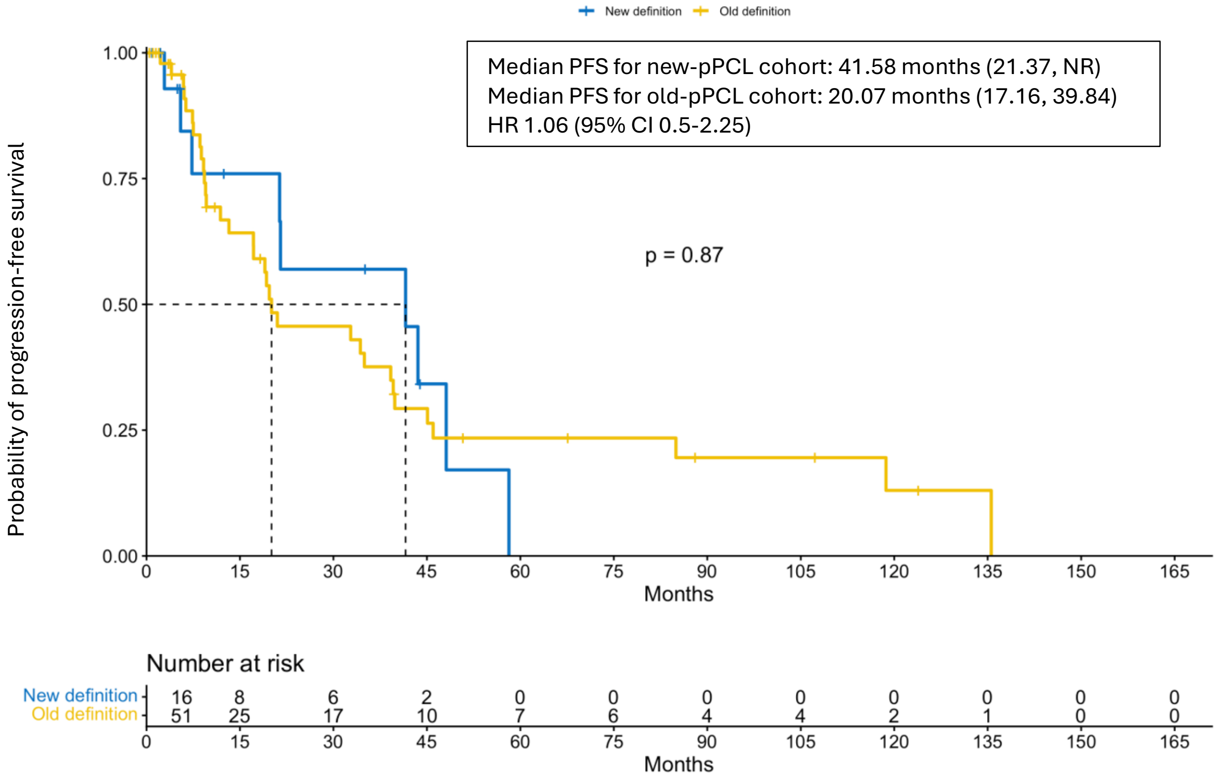

Supplementary Figure S1. KM for progression free survival comparing old-pPCL and new-pPCL cohort.
